# Respiratory virus surveillance in hospitalized children less than two-years of age in Kenema, Sierra Leone during the COVID-19 pandemic (October 2020- October 2021)

**DOI:** 10.1371/journal.pone.0292652

**Published:** 2023-10-10

**Authors:** Robert J. Samuels, Ibrahim Sumah, Foday Alhasan, Rendie McHenry, Laura Short, James D. Chappell, Zaid Haddadin, Natasha B. Halasa, Inaê D. Valério, Gustavo Amorim, Donald S. Grant, John S. Schieffelin, Troy D. Moon

**Affiliations:** 1 Kenema Government Hospital, Ministry of Health and Sanitation, Kenema, Sierra Leone; 2 Department of Pediatrics, Division of Pediatric Infectious Diseases, Vanderbilt University Medical Center, Nashville, Tennessee, United States of America; 3 Vanderbilt Institute for Global Health, Vanderbilt University Medical Center, Nashville, Tennessee, United States of America; 4 Department of Biostatistics, Vanderbilt University Medical Center, Nashville, Tennessee, United States of America; 5 College of Medicine and Allied Health Sciences, University of Sierra Leone, Freetown, Sierra Leone; 6 Department of Pediatrics, Division of Pediatric Infectious Diseases, Tulane University, New Orleans, Louisiana, United States of America; 7 Department of Tropical Medicine, Tulane University School of Public Health and Tropical Medicine, New Orleans, Louisiana, United States of America; The University of Hong Kong, CHINA

## Abstract

Globally, viral pathogens are the leading cause of acute respiratory infection in children under-five years. We aim to describe the epidemiology of viral respiratory pathogens in hospitalized children under-two years of age in Eastern Province of Sierra Leone, during the second year of the SARS-CoV-2 pandemic. We conducted a prospective study of children hospitalized with respiratory symptoms between October 2020 and October 2021. We collected demographic and clinical characteristics and calculated each participant´s respiratory symptom severity. Nose and throat swabs were collected at enrollment. Total nucleic acid was purified and tested for multiple respiratory viruses. Statistical analysis was performed using R version 4.2.0 software. 502 children less than two-years of age were enrolled. 376 (74.9%) had at least one respiratory virus detected. The most common viruses isolated were HRV/EV (28.2%), RSV (19.5%) and PIV (13.1%). Influenza and SARS-CoV-2 were identified in only 9.2% and 3.9% of children, respectively. Viral co-detection was common. Human metapneumovirus and RSV had more than two-fold higher odds of requiring O2 therapy while hospitalized. Viral pathogen prevalence was high (74.9%) in our study population. Despite this, 100% of children received antibiotics, underscoring a need to expand laboratory diagnostic capacity and to revisit clinical guidelines implementation in these children. Continuous surveillance and serologic studies among more diverse age groups, with greater geographic breadth, are needed in Sierra Leone to better characterize the long-term impact of COVID-19 on respiratory virus prevalence and to better characterize the seasonality of respiratory viruses in Sierra Leone.

## Introduction

A majority of school absenteeism, hospitalizations, and deaths in children under-five years of age in Africa is attributable to acute respiratory infections (ARI) [1, 2]. To date, much of the focus for ARI in developing countries has been on identifying bacterial causes such as *Streptococcus pneumoniae* and *Haemophilus influenzae* type b *(Hib)* in this age group [1, 3–7]. However, many low-resource settings lack the basic diagnostic capacity for identification of such bacterial and/or viral respiratory infections. As a result, a certain proportion of children admitted to hospital with presumed bacterial infection will fail antibiotic treatment, leaving clinicians frustrated and questioning their treatment decisions. Moreover, widespread antibiotic use in this population is likely associated with increased risk for the promotion of antimicrobial resistance [5, 8].

Globally, viral pathogens are now the leading cause of ARI, especially after the introduction of *Hib* and pneumococcal conjugate vaccines. Further, recent studies from several African countries have also identified viral pathogens as key contributors of ARI in children under five-years of age [7, 9, 10]. As the number of immunization programs for the common bacterial causes of ARI increases across sub-Saharan Africa, the proportion of ARI caused by respiratory viral pathogens has increased [1]. In these cases, viral ARI may cause symptoms alone or be associated with a co-infection with other viruses or bacteria, leading to severe pneumonia [1, 9]. The COVID-19 pandemic has been associated with changing trends in the prevalence of other respiratory viruses, possibly as a result of public health measures aimed at limiting circulation of SARS-CoV-2. However, this phenomenon is not yet fully understood and warrants continued vigilance as COVID-19 mitigation strategies are relaxed around the world [11–16].

Sierra Leone, located in West Africa has an estimated population of 8.6 million people as of 2022 and was ranked 182 out of 189 countries on the United Nations Development Program (UNDP) Human Development Index (HDI) for the same year [17, 18]. Health infrastructure was severely affected by civil war (1991–2002), and then again by the West African Ebola Outbreak (2013–2015) [19, 20]. Since 2006, Sierra Leone has established national-level influenza surveillance and began contributing to the World Health Organization (WHO) Strengthening Influenza Sentinel Surveillance in Africa (SISA) network in 2011 [21, 22]. Despite this, there remains a paucity of information in Sierra Leone related to the prevalence, temporality, and epidemiology of the most common and emerging human respiratory viruses.

The major aim of this study is to describe the prevalence and temporality of viral respiratory pathogens in hospitalized children under-two years of age, at one rural hospital in Eastern Province of Sierra Leone, during the second year of the SARS-CoV-2 pandemic and to characterize the social, environmental, and medical risk factors associated with severe disease.

## Materials and methods

### Study design and population

We conducted a prospective surveillance study of children admitted to the Kenema Government Hospital (KGH) pediatric ward between October 2020 and October 2021. Children were eligible for enrollment if they were <24 months of age, had been admitted to the hospital within 48-hours prior to enrollment, with or without fever of ≥ 38.0°C, and presented with at least one of the following respiratory symptoms: cough (<14 days duration); difficulty breathing (retractions, tachypnea for age: >50 breaths per minute for those 0–12 months of age and >40 breaths per minute for those 13–23 months of age), and/or nasal flaring. Patients who did not meet the above criteria were excluded from participation. Participants were only enrolled in the study once.

### Study setting

This study was conducted at KGH, in Kenema District of Eastern Province, Sierra Leone. KGH has a catchment area endemic for illnesses such as malaria and Lassa fever (LF) and is a hospital that was at the epicenter of Sierra Leone´s efforts during the 2014–2016 West African Ebola outbreak [19, 23, 24]. The pediatric ward of KGH provides free health care for children under five years of age and admits approximately 400 children per month. The ward uses the Emergency Triage, Assessment and Treatment plus (ETAT+) protocol for the management of patients. For example, children with oxygen saturation less than 90% are initiated on supplemental oxygen therapy. The ward has a unit that serves both as an emergency room and an intensive care unit. Admission to this unit is for patients with emergency and some priority signs as per the ETAT+ protocol [25]. Malaria, pneumonia, malnutrition, and gastrointestinal illness are the four main reported causes of hospitalization in the under-five age group. KGH has radiograph capacity on site but lacks both blood culture capacity and the ability to diagnose respiratory viral pathogens. Routine laboratory analysis is limited to simple hematology and chemistries; rapid tests for malaria, HIV, typhoid, and syphilis; microscopy for malaria and stool samples; as well as point of care technologies such as GeneXpert MTB/RIF (Cepheid: Sunnyvale, CA) for tuberculosis (TB).

### Recruitment

Upon admission, children with acute respiratory symptoms were pre-screened for potential eligibility by emergency care nurses and the hospital admission team. Once preliminary eligibility was established, parents or legal guardians were approached by trained study personnel for enrollment until a daily quota was met (approximately three new patients enrolled per day). Enrollment took place Monday-Friday during daytime work hours (6am to 6pm).

### Data collection

Data were collected by study nurses utilizing a paper-based study instrument and then uploaded into a password-protected, tablet-based, online Research Electronic Data Capture (REDCap) database (www.project-redcap.org). Parents or legal guardians were asked a series of questions using a standardized case report form (CFR). The CFR covered seven broad areas: Introduction and screening, demographics and social history, current illness, diet/nutrition, physical examination, and laboratory results. At admission, participants´ respiratory symptom severity was recorded using an adapted version of the bronchiolitis severity score published by Tal et al, with patients subsequently categorized as having mild, moderate or severe symptoms [26]. Chart reviews were done during admission and upon discharge, referral, or death. This method allowed for the recording of demographic information, medical and medication history, and information on clinical course while hospitalized. Data quality control was conducted by study investigators who reviewed all completed paper-based study instruments and confirmed the accuracy of data entered into the electronic database.

### Laboratory procedures

Nose and throat swabs were collected by the study team at enrollment and combined immediately in viral transport medium. Samples were labeled with the participant’s identification number to ensure anonymity and stored at -80°C (KGH) until shipment on dry ice (Nov. 2021) to Vanderbilt University Medical Centre (VUMC) for testing.

Total nucleic acid (TNA) was purified from aliquoted specimens using the QIAamp 96 Virus QIAcube HT Kit and QIAcube HT automated extraction system (QIAGEN, Germantown, MD) and maintained at -80°C until analysis. TNA extracts were tested for the following pathogen targets using the *NxTAG Respiratory Pathogen Panel + SARS-CoV-2* (Luminex, Austin, TX): adenovirus (AdV); human endemic coronaviruses (hCoV) 229E, HKU1, NL63, OC43; SARS-CoV-2; human bocavirus (hBoV); human metapneumovirus (hMPV); influenza A and subtypes 2009 H1N1, H1, and H3; influenza B and influenza C; parainfluenza (PIV) 1–4; respiratory syncytial virus (RSV) A and B; human rhinovirus/enterovirus (HRV/EV); *Chlamydophila pneumoniae*; and *Mycoplasma pneumoniae*. Reverse-transcription quantitative PCR (RT-qPCR) on the QuantStudio 3 and QuantStudio 6 Pro Real Time PCR Systems was employed to test extracts for the following targets using *SuperScript III One-Step RT-PCR System* with current versions of assays developed at the US Centers for Disease Control and Prevention (*CDC Human Influenza Virus Real-Time RT-PCR Diagnostic Panel Influenza A/B Typing Kit*, *Influenza A Subtyping Kit*, and *Influenza B Lineage Genotyping Kit*; and *Influenza C Assay*): influenza A and subtypes, influenza B and lineage genotypes, influenza C, and human RNase P (endogenous target for specimen adequacy).

### Statistical analysis

Descriptive statistics were presented as frequency (percentage) or median and interquartile range [IQR] where appropriate. Categorical variables were compared using Pearson χ^2^ tests. Continuous variables were compared using Mann-Whitney U test. Multivariable logistic regression was used to find risk factors associated with higher odds of being referred to the intensive care unit (ICU) or requiring oxygen (O2) during hospitalization. Variables included in the final model were pre-specified and selected based on literature review and clinical knowledge. In order to avoid subjective discretization of a continuous variable, we analyzed severity score as an ordinal, continuous variable, and used a multivariable ordinal regression model with a logit link to regress it on the same covariates used in both logistic regressions mentioned above [27]. Data discretization refers to a process of converting large amounts of data values into smaller ones, so that their subsequent evaluation and management becomes easier. Continuous variables, such as age and birthweight, were modelled using restricted cubic splines, with three knots equally spaced to alleviate linearity assumptions. Interpretation of these non-linear effects on the outcomes used, contrasts between pre-specified age and BMI, as suggested by Shepherd and Rebeiro (2017) [28]. A propensity score approach was used to estimate the effect of each type of virus on all three outcomes (being referred to ICU, requiring O2, and severity score). More specifically, we defined a binary variable to identify patients that were diagnosed with a particular virus of interest. For instance, while focusing on AdV, patients that were diagnosed with AdV would be label as “1” while patients that did not have AdV detected in their samples received a value of “0”. The propensity score, i.e., the probability of being diagnosed with AdV was estimated via multivariable logistic regression, adjusting for the following baseline covariates: age, sex, birthweight, and number of people living in the same household. Continuous variables were modelled using restricted cubic splines, with three knots equally spaced. The estimated propensity score was then included as a covariate in the outcome model, which regressed the outcome on the type of virus of interest. This propensity score approach was repeated for each type of virus [29]. Statistical analysis was performed using R version 4.2.0 software.

### Ethics statement and inclusivity in global research

This project was approved by the Sierra Leone Ethics and Scientific Review Committee (SLESRC April 3, 2020) and the Institutional Review Board at Vanderbilt University Medical Center (IRB# 201203). Written informed consent was obtained from the parent or legal guardian of all children enrolled in the study.

Additional information regarding the ethical, cultural, and scientific considerations specific to inclusivity in global research is included in the Supporting Information (S1 Checklist).

## Results

### Study population

From October 01, 2020 to October 31, 2021 a total of 1,226 children were screened with 695 confirmed eligible per criteria. Of these, 166 were not enrolled due to parent/guardian refusal. A total of 502 children less than 2-years of age were enrolled and included in this analysis.

### Demographic and clinical characteristics of hospitalized children

Of the 502 children enrolled, 239 (47.6%) were female with a median age of 8.6 months [IQR: 4.8; 14.2] (Table 1). Maternal education was low, with only 53.2% having achieved greater than a 6^th^ grade level (primary education or less). Median household size was 8.0 persons [IQR: 5.0; 12.0] and the median number of children residing in each household that were less than two years of age was 2.0 [IQR 1.0; 3.0]. About 38.6% of parents reported that another household member also had an ARI in the two weeks prior to their child’s admission to hospital. 39.6% of households reported having a smoker living in the household and only 32 households (6.4%) reported using an in-door wood burning stove.

**Table 1:** Demographic and clinical characteristics of children under two-years hospitalized with respiratory symptoms, Kenema, October 2020-October 2021.

Variables (N = 502)	N (%)
**Sociodemographic characteristics**	
Sex	
Female	239 (47.6%)
Age in months **(median [IQR])**	8.6 [4.8;14.2]
Mother’s education	
No education	166 (33.1%)
Primary education	69 (13.7%)
Secondary education	218 (43.4%)
Some or completed college	49 (9.8%)
Number of household members, **(median [IQR])**	8.0 [5.0;12.0]
Number in household members aged < 5 years **(median [IQR])**	2.0 [1.0;3.0]
Other household member with acute respiratory infection in 2 weeks prior to admission	194 (38.6%)
Smoker lives in the household	199 (39.6%)
Household uses an indoor burning stove	32 (6.4%)
**Birth characteristics**	
Premature, <37 weeks	344 (68.5%)
Cesarean section	73 (14.5%)
Birth weight in kilograms, **(median [IQR]) (n = 501)**	3.0 [2.8;3.4]
**Nutrition characteristics**	
Currently breastfeeding	385 (76.7%)
Exclusive breastfeeding	91 (23.6%)
Supplementing with formula	209 (54.3%)
Supplementing with solid foods	85 (22.1%)
Duration of breastfeeding in months, **(median [IQR]) (n = 491)**	8.0 [5.0;13.0]
Acute Malnutrition* **(n = 498)**	
Moderate (MAM)	88 (17.7%)
Severe (SAM)	98 (19.7%)
Global (GAM)	186 (37.3%)
Chronic Malnutrition**(n = 500)	
Moderate (MCM)	84 (16.8%)
Severe (SCM)	113 (22.6%)
Global (GCM)**	197 (39.4%)
**Diagnosis at admission**	
No respiratory virus detected (n = 502)	126 (25.1%)
Respiratory virus detected^&^	376 (74.9%)
≥1 Respiratory viruses detected (n = 376)	97 (25.7%)
Adenovirus (AdV)	37 (7.4%)
Human Bocavirus (hBoV)	26 (5.2%)
Endemic Human Coronaviruses (hCoV)	28 (5.6%)
Human metapneumovirus (hMPV)	24 (4.8%)
Influenza (A, B, C)	46 (9.2%)
Parainfluenza viruses (PIV 1–4)	66 (13.1%)
Human Rhinovirus/Enterovirus (HRV/EV)	142 (28.2%)
Respiratory Syncytial Virus (RSV)	98 (19.5%)
SARS-CoV-2	20 (3.9%)
Other diagnosis and/or treatment	
Malaria (positive rapid diagnostic test or blood smear result)	213 (42.4%)
Antimalarials given	292 (58.2%)
Malaria + viral co-infection (n = 213)	147 (69.0%)
HIV/AIDS test performed	66 (13.1%)
HIV/AIDS: positive	2 (3.0%)
Tuberculosis	7 (13.1%)
Sickle cell test performed	36 (7.2%)
Sickle cell: positive	7 (19.4%)
Antibiotics given prior to hospitalization with this illness	177 (35.3%)
Antibiotics given during hospitalization	502 (100.0%)
**Respiratory symptom severity**	
Mild	403 (80.2%)
Moderate	86 (17.1%)
Severe	13 (2.5%)
Received Supplemental Oxygen	170 (33.8%)
Referred to intensive care unit (ICU)	303 (60.3%)
** Status at discharge**	
Alive	459 (91.4%)
Dead	24 (4.8%)
Discharged against medical advice/abscond	19 (3.8%)
Length of stay in hospital in days **(median [IQR])**	3.0 [2.0: 6.0]

*Moderate acute malnutrition = weight for height z-score between -2 and -3

*Severe acute malnutrition = weight for height z-score > -3

*Global acute malnutrition = weight for height z-score > -2 (moderate + severe)

**Moderate Chronic malnutrition = height for age z-score between -2 and -3

**Severe Chronic malnutrition = height for age z-score > -3

**Global Chronic malnutrition = height for age z-score > -2 (moderate + severe)

^&^Of 376 participants with a viral infection detected, a total of 487 viruses were isolated. Percentages of virus’ represents the proportion of a given virus out of the 502 total number of participants.

Of the enrolled children, more than half were born premature (<37 weeks gestational age) (344, 68.5%) and the median birth weight of the enrolled children was 3.0 kilograms [IQR: 2.8; 3.4]. Seventy-three (14.5%) children enrolled were born by cesarean section. At the time of enrollment, 385 (76.7%) children reported that their diet still consisted of breastfeeding, of which 91 (23.6%) were exclusively breastfed, 209 (54.3%) were supplementing with formula feeds, and 85 (22.1%) were supplementing with solid foods. The median duration of breastfeeding was 8.0 months [IQR: 5.0; 13.0]. Malnutrition was common, with 186 children (37.3%) classified as having global acute malnutrition (weight for height z-score > -2) and 197 children (39.4%) classified as having global chronic malnutrition (height for age z-score > -2).

Human immunodeficiency virus (HIV), tuberculosis (TB), and sickle cell disease were uncommon in these children; however, 213 (42.4%) had a laboratory confirmed malaria diagnoses at the time of admission. Prior to hospitalization 35.3% of children had received antibiotics for this illness and 100% of children were administered antibiotics during their hospital stay. The median length of hospital stay was 3.0 days [IQR: 2.0; 6.0] and almost all (91.4%) were alive at discharge from the hospital.

### Respiratory virus detection

**Fig 1:**
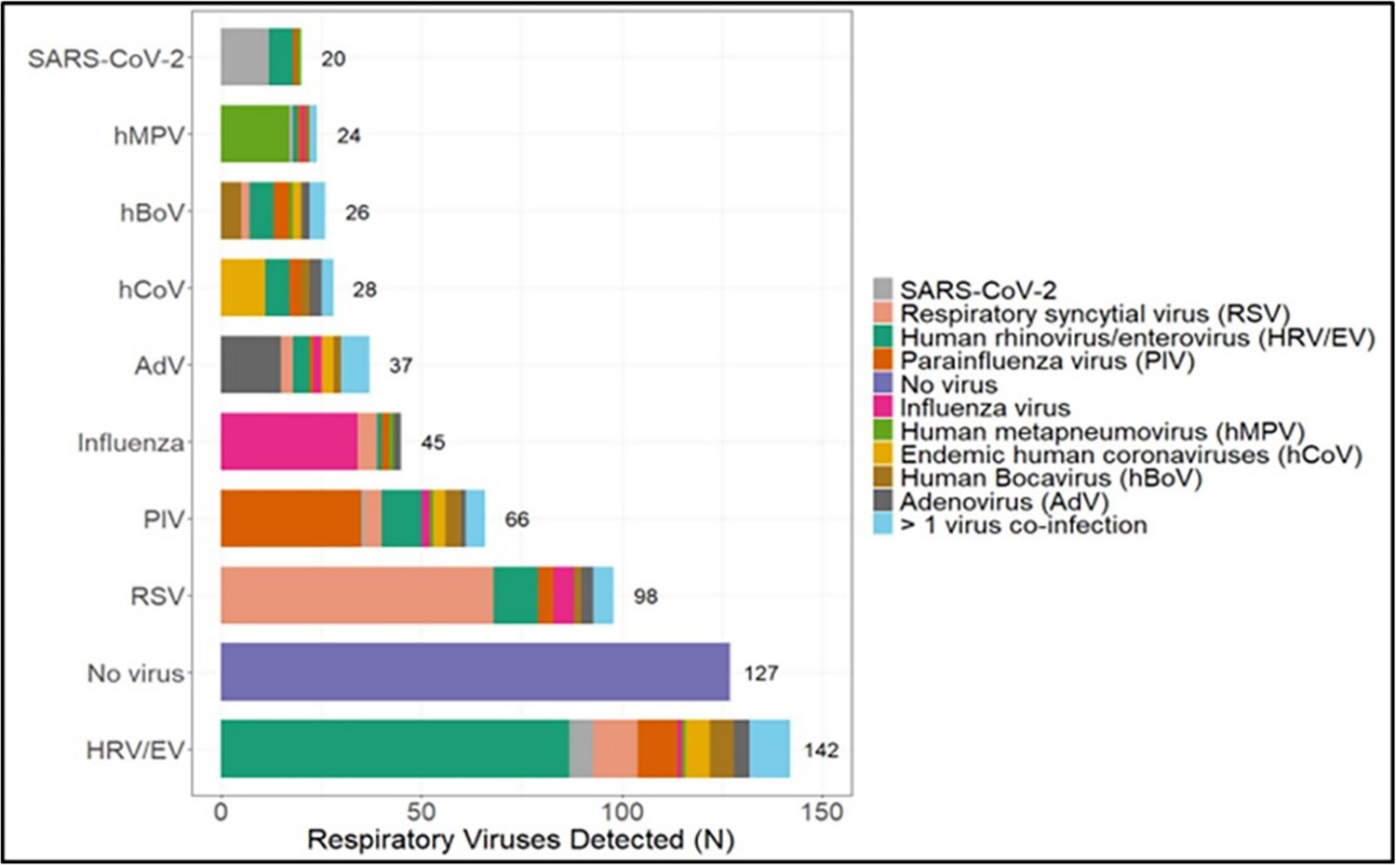
Total number of viral pathogens identified and their associated co-detected viruses.

Of our 502 enrolled children, 376 (74.9%) had at least one respiratory virus detected by nasal swab testing with PCR. The most common virus isolated was HRV/EV (28.2%), followed by RSV (19.5%) and PIV (13.1%). Influenza and SARS-CoV-2 were identified in only 9.2% and 3.9% of children, respectively. Viral co-detection was common, with 97/376 (25.7%) having more than one virus identified (Fig 1). Of the 213 children with a laboratory confirmed malaria diagnosis, 147 (69.5%) had a virus identified as well.

The demographic and clinical characteristics of enrolled children were compared based on viral detection by PCR testing (Table 2). Children with a virus detected were younger (median age 8.13 months [IQR: 4.47; 13.70] vs. 10.5 months [IQR: 6.15; 15.60]; p = 0.009); had a shorter duration of reported breastfeeding (median 7.0 months [IQR 4.0; 12.0] vs. 9.5 months [IQR: 5.0; 14.0], p = 0.025); were less frequently classified as having global chronic malnutrition (36.5% vs. 48.0%, p = 0.030); and had a lower frequency of malaria co-infection at the time of admission (39.4% vs. 51.6%, p = 0.022).

**Table 2:** Demographic and clinical characteristics of children under two-years, by whether a virus was detected on PCR, hospitalized with respiratory symptoms, Kenema, October 2020-October 2021.

Variables (N = 502)	Virus-positive 376 (75%)	Virus-negative 126 (25%)	p-value
**Sociodemographic characteristics**			
Sex			0.605
Female	176 (46.8%)	63 (50.0%)	
Age in months **(median [IQR])**	8.13 [4.47;13.7]	10.5 [6.15;15.6]	**0.009**
Mother’s education			0.338
No education	118 (31.4%)	48 (38.1%)	
Primary education	52 (13.8%)	17 (13.5%)	
Secondary education	165 (43.9%)	53 (42.1%)	
Some or completed college	41 (10.9%)	8 (6.4%)	
Number of household members, **(median [IQR])**	8.0 [5.0;12.0]	8.0 [5.0;11.]	0.724
Number of household members aged < 5 years **(median [IQR])**	2.0 [1.0;3.0]	2.00 [1.0;2.0]	0.112
Other household member with acute respiratory infection in 2-weeks prior to admission	146 (38.8%)	48 (38.1%)	0.967
Smoker lives in the household	149 (39.6%)	50 (39.7%)	0.493
Household uses an indoor burning stove	24 (6.4%)	8 (6.4%)	1.000
**Birth characteristics**			
Premature, <37 weeks	262 (69.7%)	82 (65.1%)	0.394
Cesarean section	51 (13.6%)	22 (17.5%)	0.354
Birth weight in kilograms, **(median [IQR]) (n = 501)**	3.0 [2.8;3.4]	3.0 [2.7;3.3]	0.344
**Nutrition characteristics**			
Duration of breastfeeding in months, **(median [IQR]) (n = 491)**	7.0 [4.0;12.0]	9.5 [5.0;14.0]	**0.025**
Global Acute Malnutrition (GAM)* **(n = 498)**	135 (36.2%)	51 (41.1%)	0.381
Global Chronic Malnutrition (GCM)** **(n = 500)**	137 (36.5%)	60 (48.0%)	0.030
**Other diagnosis and/or treatment**			
Malaria (positive rapid diagnostic test or blood smear result)	148 (39.4%)	65 (51.6%)	**0.022**
Antimalarials given	212 (56.4%)	80 (63.5%)	0.195
Antibiotics given prior to hospitalization with this illness	140 (37.2%)	37 (29.4%)	0.136
Antibiotics given during hospitalization	376 (100%)	126 (100%)	1.001
**Status at discharge**			0.492
Alive	344 (91.5%)	115 (91.3%)	
Dead	17 (4.5%)	7 (5.6%)	
Discharged against medical advice/abscond	15 (3.9%)	4 (3.2%)	
Length of Stay in Hospital in Days	4.0 [2.0;6.0]	3.0 [2.0;5.0]	0.388

*Global Acute Malnutrition = weight for height z-score > -2

**Global Chronic Malnutrition = height for age z-score > -2

### Seasonality of respiratory viruses

**Fig 2:**
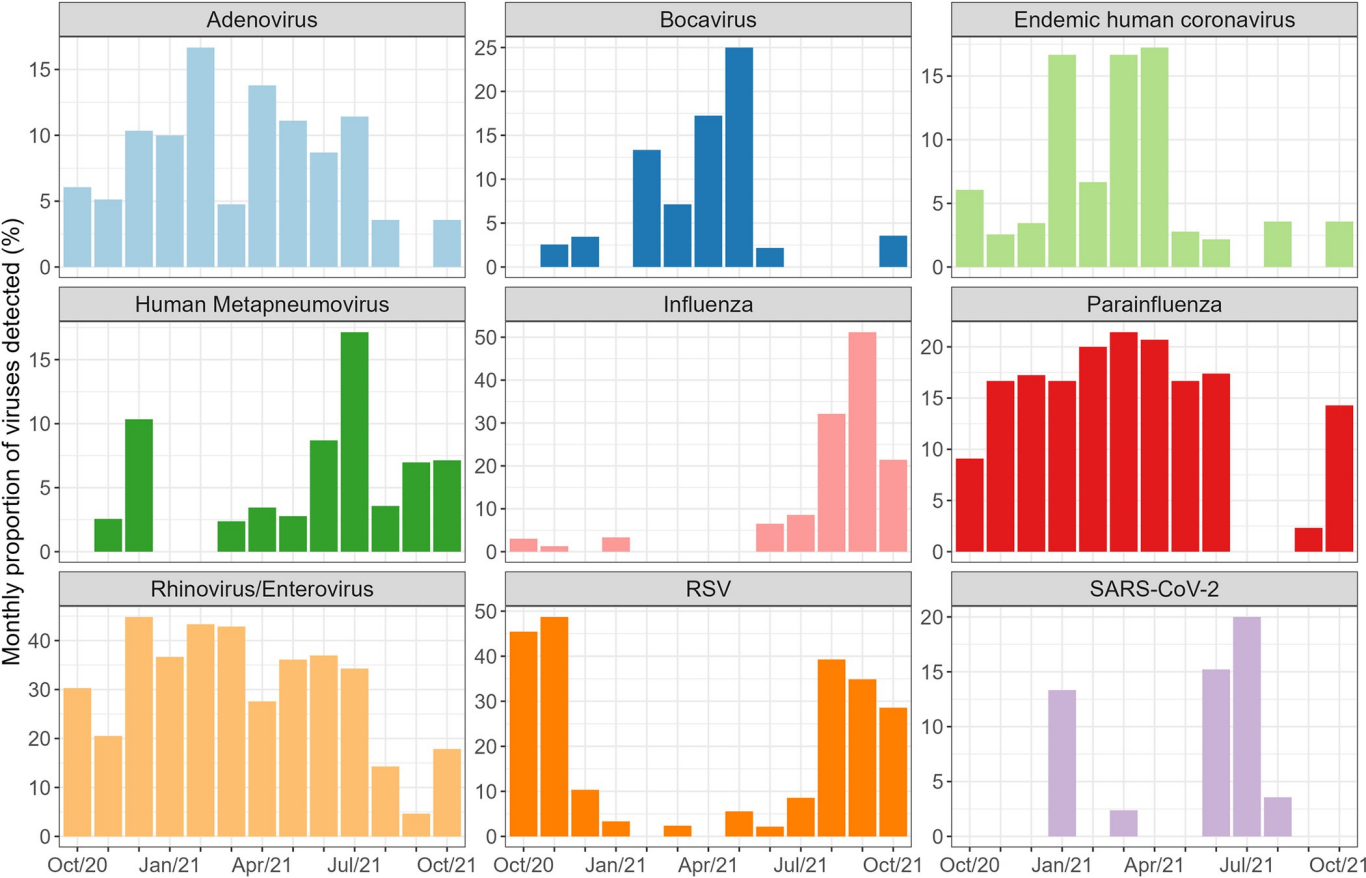
Respiratory virus detection by month (October 2020 to October 2021).

HRV/EV, AdV, and PIV all had a fairly steady year-round circulation with only a slight decline in cases seen between August to October. All other viruses showed more distinct seasonal peaks (Fig 2). For example, the endemic common cold hCoV’s and hBoV showed peaks in cases between January and April, during a time that corresponds with Sierra Leone’s typical dry season (December to April). In contrast, influenza, RSV, and hMPV showed a peak in cases that corresponded more to Sierra Leone’s wet season (May to November), with hMPV appearing earlier in June and July, while RSV and influenza peaked later from August to November.

Overall, the number of cases of SARS-CoV-2 in our population was low (only 20 total), despite the fact that enrollment into our study corresponded to a period of intense pandemic escalation. In our patients, SARS-CoV-2 detection was predominantly limited to January 2021 and a second peak in June and July 2021.

### Determinants of need for O2 therapy, referral to the ICU, and increased severity score

In multivariable analysis, we explored patient characteristics associated with the need for O2 therapy administration, the need for referral to a higher level of care in the ICU, and their impact on severity score (Table 3). Children who were of younger age (6 months vs. 12 months of age) had a roughly two-fold higher likelihood of requiring O2 therapy (aOR 2.02, 95%CI:1.57, 2.58, p = 0.007) than older children. Similar results were observed for referral to the ICU and severity scores, although the overall effect of age in both regressions was not statistically significant (p>0.05); younger patients (6 months compared to 12 months) were more likely to require ICU (aOR 1.64, 95%CI:1.24, 2.18) and to have higher severity score (aOR 1.86, 95%CI: 1.51, 2.28). Further, children with more than one virus detected had an approximately 1.7-fold higher odds of requiring O2 therapy during their hospitalization compared to those who had a single virus detected. For children with malnutrition, we saw mixed results. Children classified as having Global Acute Malnutrition had a 61% lower odds of being transferred to the ICU (aOR 0.39, 95%CI: 0.26, 0.61, p<0.001), while those classified as having Global Chronic Malnutrition had 53% lower odds of being transferred to the ICU (aOR 0.47, 95%CI: 0.30, 0.72, p = 0.001). Children that received antibiotics prior to admission had also higher odds of having higher severity scores (aOR 1.41, 95%CI: 1.01, 1.98, p = 0.042). We also performed sensitivity analysis to assess the effect of multicollinearity between acute and chronic malnutrition. We ran additional regression models, adjusting for each variable at a time, and assessed its effect on the outcomes of interest. The results were the same as when both variables were included simultaneously in the models and are thus omitted from presentation.

**Table 3:** Multivariable logistic regression for variables associated with need for oxygen, referral to the ICU, and increasing severity score.

	O2 required		Referral to ICU		Severity Score	
Variables	aOR (95% CI)	p-value	aOR (95% CI)	p-value	aOR (95% CI	p-value
Age		0.007		0.187		0.064
6 months	2.02 (1.57, 2.58)		1.64 (1.24, 2.18)		1.86 (1.51, 2.28)	
12 months	Ref		Ref		Ref	
18 months	0.95 (0.64,1.41)		0.85 (0.59, 1.22)		0.77 (0.57, 1.03)	
Male	0.71 (0.47, 1.08)	0.110	1.13 (0.75, 1.72)	0.558	0.91 (0.19, 4.35)	0.553
Prematurity (<37 weeks)	1.33 (0.85, 2.08)	0.206	1.30 (0.84, 2.02)	0.247	1.21 (0.72, 2.03)	0.287
Virus co-infection (more than one virus)	1.71 (1.04, 2.80)	0.033	0.69 (0.41, 1.14)	0.145	1.18 (0.53, 2.63)	0.419
Exposure to indoor pollutants*	1.17 (0.78, 1.75)	0.462	0.81 (0.54, 1.22)	0.308	1.24 (0.90, 1.72)	0.186
Global Acute Malnutrition^&^	1.27 (0.83, 1.94)	0.276	0.39 (0.26, 0.61)	<0.001	1.22 (0.88, 1.68)	0.251
Global Chronic Malnutrition^@^	0.82 (0.52, 1.27)	0.370	0.47 (0.30, 0.72)	0.001	0.79 (0.56, 1.13	0.187
Length of stay in Hospital in days	1.04 (0.99, 1.08)	0.104	0.99 (0.96, 1.02)	0.496	1.01 (0.67, 1.52)	0.331
Antibiotics received prior to admission	1.16 (0.76, 1.77)	0.478	0.74 (0.48, 1.13)	0.160	1.41 (1.01, 1.98)	0.042

Ref: Reference level

^&^ Global Acute Malnutrition = weight for height z-score > -2

^@^Global Chronic Malnutrition = height for age z-score > -2

*Exposure to indoor pollutants: People living with a smoker and use of an indoor burning stove

When we explored associations between virus type and need for O2 therapy, ICU referral, or increased severity scores, we found both hMPV (aOR 2.61, 95%CI:1.09, 6.21, p = 0.029) and RSV (aOR 2.03, 95%CI:1.26, 3.27, p = 0.003) had more than two-fold higher odds of requiring O2 therapy while hospitalized (Table 4). No associations were found between virus type and referral to the ICU. Influenza was associated with lower severity scores (aOR 0.58, 95%CI: 0.35, 0.99 p = 0.045). Although, on average, patients with RSV had higher odds of increased severity scores (OR 1.39, 95%CI: 0.93, 2.07, p = 0.106), this association was not statistically significant (p>0.05).

**Table 4:** Multiple logistic regression with propensity score adjustment for the association between virus and worsening clinical condition.

	O2 required		Referred to ICU		Severity Score	
Viruses	aOR (95% CI)	p-value	aOR (95% CI)	p-value	aOR (95% CI)	p-value
AdV	1.20 (0.58, 2.47)	0.615	0.56 (0.28, 1.12)	0.100	0.93 (0.51, 1.68)	0.799
hBoV	0.80 (0.34, 1.90)	0.620	0.50 (0.22, 1.12)	0.090	1.03 (0.51, 2.10)	0.932
hCoV	1.04 (0.46, 2.37)	0.916	1.21 (0.54, 2.71)	0.633	1.22 (0.62, 2.40)	0.569
SARS-CoV-2	2.10 (0.85, 5.02)	0.108	0.65 (0.26, 1.61)	0.360	1.85 (0.79, 4.33)	0.157
Influenza A, B, and C	0.69 (0.34, 1.42)	0.318	1.26 (0.66, 2.43)	0.477	0.58 (0.35, 0.99)	0.045
hMPV	2.61 (1.09, 6.21)	0.029	2.11 (0.75, 5.87)	0.151	1.18 (0.57, 2.43)	0.661
PIV 1–4	1.07 (0.61, 1.87)	0.803	0.89 (0.52, 1.52)	0.684	1.33 (0.83, 2.13)	0.235
HRV/EV	0.87 (0.58,1.32)	0.519	0.98 (0.65, 1.46)	0.936	0.97 (0.69, 1.37)	0.882
RSV	2.03 (1.26, 3.27)	0.003	0.81 (0.50, 1.32)	0.410	1.39 (0.93, 2.07)	0.106

AdV = adenovirus; hBoV = Human Bocavirus; hCoV = Human endemic coronaviruses; hMPV = Human metapneumovirus; PIV = parainfluenza virus; HRV/EV = Human rhinovirus/enterovirus; RSV = respiratory syncytial virus.

Propensity score model adjusted for age, gender, birthweight, and number of persons living in the household.

## Discussion

Repeated global outbreaks of respiratory virus infections have had negative impacts on overall population-level health as well as placing significant strains on the global economy and national development efforts [30]. The recent pandemic of severe acute respiratory syndrome coronavirus 2 (SARS-CoV-2) and associated novel coronavirus disease 2019 (COVID-19) highlight this point and underscore the urgency for strengthening global public health surveillance for early detection and control of respiratory viruses.

Our study describes the prevalence and temporality of viral respiratory pathogens identified in children less than 24 months of age who were admitted to hospital between October 2020 and October 2021 in rural eastern Sierra Leone with acute respiratory symptoms. This period corresponds to the latter part of the first year of the COVID-19 pandemic and much of the pandemic’s second year. Viral infection incidence and temporality are impacted by a variety of factors including precipitation; temperature and humidity; hemisphere location; as well as human behavior and the impact of changing environmental conditions on host defense mechanisms [31, 32]. To date, quite a bit is known about the epidemiology and seasonal variation of viruses in temperate climates. For example, influenza, RSV and the endemic hCoV’s are well described to typically occur in the colder, dryer winter months in temperate climates, while viruses such as PIV, hMPV, and HRV/EV are more likely to peak during the spring and early summer months [32–34]. In contrast, a much smaller number of studies have reported on the seasonal variation of respiratory viruses in tropical countries [7, 32, 35]. As such, one of the goals of our study was to provide baseline data on respiratory virus prevalence among a younger pediatric population, in a tropical region of West Africa, in order to aid health authorities with decision making around surveillance and prevention.

In our study, nearly three-quarters of the children had at least one virus detected. This is comparable to many other surveillance studies both in developed and developing countries. The overall prevalence of these viruses is also comparable to previous studies which have highlighted RSV, PIV, influenza, and hMPV as the most common viral etiologies of ARI among children in the under-five year age group [33, 36, 37].

HRV/EV and PIV’s were two of the top three viruses detected in our study population. Together with AdV, which was detected in 7.4% of children, and the endemic hCoV’s, which were detected in 5.6%, these viruses circulated mostly year-round, without distinct seasonal variability. Each of these viruses, however, showed a distinct decline in cases corresponding with the main peak in influenza cases in our study (August to October 2021). Respiratory virus interference, in which respiratory virus transmission can interfere or inhibit the transmission of other respiratory viruses, has been previously described, especially in relation to peaks in influenza cases and may have contributed to the pattern of seasonal variability seen in our study population [38–40].

Multiple reports have described that infection with a respiratory virus generally follows a recognizable seasonal pattern in temperate climates, whereas they may circulate all year round in tropical regions [41–44]. In these studies, peaks in RSV and influenza cases have tended to correspond to winter months in temperate regions, while showing greater diversity in temporality in tropical regions. Furthermore, peaks in RSV and influenza cases have occurred later in the year, comparatively, as geographic latitude increases. In tropical countries that have distinct wet and dry seasons, the peak in cases of RSV and influenza have tended to correspond mostly with the wet, rainy season [7, 36, 45]. In Sierra Leone, the wet season occurs each year during the period from May to November, with the heaviest rain fall typically between July and September. This is then followed by a dry season from December to April [46]. During our period of study, this predilection for cases peaking in the wet season was similarly observed, with RSV cases peaking between October and November in 2020. Then in 2021, both RSV and influenza cases peaked during the heaviest rain fall months between August and October. It has been well documented that there were reductions in the annual epidemics of most seasonal respiratory viruses in 2020/2021, likely as a result of the COVID-19 pandemic and the large scale strategies implemented worldwide that year in an attempt to mitigate COVID-19 [13, 47, 48]. Influenza in particular had a notable absence globally during the 2020/2021 seasons, and in the southern hemisphere in particular, showed very low levels of activity from May to September 2020 during the southern hemisphere´s typical winter months [12, 49]. While our study period only catches the last few months of Sierra Leone´s typical 2020 influenza period, the absence of influenza cases in 2020 appears to have been similar in our study population.

Children enrolled in our study were younger (median age 8.6 months) and had a high rate of respiratory virus co-infection (19.3% of all children enrolled and 25.7% among those diagnosed with any respiratory virus infection). In previous studies, younger children, especially those less than one year of age, similarly showed both higher rates of viral ARI overall as well as viral co-infection [6, 50–53]. This is likely due to a developing immune system and poor ability for viral elimination [54–56]. While data on respiratory virus co-infection remains limited, reports have described that such infections may be more severe compared to single pathogen infections [57, 58]. This was the case in our study as well, where in multivariable analysis, children with more than one virus detected had a higher odds of requiring oxygen therapy during their hospitalization (aOR 1.71, p = 0.033). Furthermore, the likelihood of needing supplemental oxygen therapy was two-fold higher in children who were younger (less than a year old) compared with older children (aOR 2.02, p = 0.007) and for children diagnosed with RSV (aOR 2.03, p = 0.003) and hMPV (aOR 2.61, p = 0.029).

Maternal education of our children was low. Studies done in comparable resource limited settings have shown similar trends in ARI frequency and disease severity in children whose mothers had lower educational attainment, possibly due to more constant physical contact between mother and child and due to the fact that knowledge of, and access to, preventive and/or therapeutic measures may be lacking [59–61]. Although this study did not show correlations between certain well-known risk factors (such as overcrowding, living with smokers in the same household, presence of indoor wood burning stove), these risk factors were documented in children presenting to the hospital with respiratory symptoms. These risk factors, although typical to these low-income settings, are modifiable, and interventions such as targeted vaccinations of vulnerable groups and continuous community engagement to improve vaccine uptake of these groups and social lifestyle could help mitigate the increased presentations of respiratory symptoms to the hospitals.

Lack of breastfeeding has been shown to put children under-five years of age at increased risk of ARI severity and hospitalizations due to the child not receiving boosted immunity from the mother [50, 62]. In our study, children with a virus detected at enrollment were more likely to have breastfed for less time (median of 7 months vs. 9.5 months; p = 0.03). In settings such as Sierra Leone, breastfeeding is already commonly practiced and significant investments have been made in the promotion of exclusive breastfeeding until six-months of age [63, 64]. Perhaps these strategies should be re-evaluated, promoting breastfeeding for longer periods of time.

Malnutrition was common in our study population, with about 37.3% and 39.4% of children categorized as having either global acute malnutrition or global chronic malnutrition respectively. Among children categorized as malnourished, more than a third were identified as having a viral infection at enrollment. Further, those categorized as having global chronic malnutrition had a 2-fold higher likelihood of being referred to the ICU during this hospitalization (aOR 2.14, p = 0.001). While we cannot determine in our study if the referral to ICU was for nutrition related issues or due to respiratory compromise or both, there is strong evidence in the literature to support the association between inadequate growth and the risk of severe disease and/or death from an acute lower respiratory infection in early childhood [65–68].

One of the key strengths of our study was having a rich data set to describe respiratory viral surveillance, in a previously unstudied pediatric population, during much of the early part of the COVID-19 pandemic. Prior to this study, no large prospective studies of respiratory viral disease had been completed in this setting. Our study, however, has some limitations as well. The KGH study site, despite being the main referral facility for sick children in Eastern Province, Sierra Leone does not receive all pediatric referrals. There are other centers with high pediatrics admissions. Furthermore, only hospitalized children were enrolled into the study, and in addition, enrollment was conducted through convenience sampling. As a result, we are unable to categorize respiratory virus prevalence among children seen in the outpatient clinics, and therefore, our results may not be a true reflection of the respiratory viral prevalence in the province, nor generalizable to the country as a whole. Our ongoing surveillance allowed us to collect samples over the course of a year, in order to begin describing the seasonality of respiratory virus prevalence in hospitalized children in this region. However, while the timing of our study allowed us a unique opportunity to describe the prevalence of respiratory viruses in young children during the global COVID-19 pandemic, we acknowledge that this also likely has had a dramatic impact on the generalizability of our results for estimating across future years. Moreover, a realistic description of the seasonal variation in respiratory virus cases more broadly, is more complicated than can be assessed in just one year. Lastly, bacterial culture capacity is non-existent in Kenema such that we were unable to analyze for bacterial causes of ARI in our patients.

## Conclusion

Viral pathogen prevalence was high (74.9%) in children under-two years of age, hospitalized with respiratory symptoms in Eastern Province, Sierra Leone. Viral co-detection with more than one virus and viral co-detection with malaria were also common. Influenza, RSV and hMPV showed peaks in cases that corresponded with Sierra Leone´s typical wet season (May to November), while the endemic common cold hCoV’s and hBoV showed peaks in cases corresponding to Sierra Leone´s typical dry season (December to April). HRV/EV, AdV, and PIV each had a fairly steady year-round circulation. Despite a high prevalence of respiratory viruses isolated, 100% of children received antibiotics, underscoring a need to expand laboratory diagnostic capacity and to revisit clinical guidelines and their implementation in the management of children presenting to low-resource facilities with respiratory symptoms. SARS-CoV-2 identification in our patients was quite low, and this finding warrants further exploration. However, the frequency of other viruses reported in our study, especially influenza, were consistent with what was reported elsewhere and may represent alterations in their seasonality as a result of the COVID-19 pandemic, though further study for longer periods of time is needed to fully elucidate these trends. Continuous surveillance and serologic studies among more diverse age groups, with greater geographic breadth, are needed in Sierra Leone to better characterize the long-term impact of COVID-19 on respiratory virus prevalence and to better characterize the seasonality of respiratory virus transmission in general in Sierra Leone.

## Supporting information

S1 Checklist This file is the inclusivity in global research checklist. (DOCX)

## References

[1] Sambala EZ, Mdolo A, Banda R, Phiri A, Wiyeh AB, Wiysonge CS. Burden of seasonal influenza in sub-Saharan Africa: a systematic review protocol. BMJ Open. 2018 10;8(10):e022949. doi: 10.1136/bmjopen-2018-022949 30309991 PMC6252638

[2] McLean HQ, Peterson SH, King JP, Meece JK, Belongia EA. School absenteeism among school-aged children with medically attended acute viral respiratory illness during three influenza seasons, 2012–2013 through 2014–2015. Influenza Other Respir Viruses. 2017 May;11(3):220–9. doi: 10.1111/irv.12440 27885805 PMC5410714

[3] Madhi SA, Klugman KP. Acute Respiratory Infections. In: Jamison DT, Feachem RG, Makgoba MW, Bos ER, Baingana FK, Hofman KJ, et al., editors. Disease and Mortality in Sub-Saharan Africa [Internet]. 2nd ed. Washington (DC): World Bank; 2006 [cited 2020 Jan 22]. Available from: http://www.ncbi.nlm.nih.gov/books/NBK2283/

[4] Loda FA, Denny FW. Acute Respiratory Infections Are the Leading Cause of Death in Children in Developing Countries. Am J Trop Med Hyg. 1986 Jan 1;35(1):1–2. doi: 10.4269/ajtmh.1986.35.1 3946732

[5] Kwofie TB, Anane YA, Nkrumah B, Annan A, Nguah SB, Owusu M. Respiratory viruses in children hospitalized for acute lower respiratory tract infection in Ghana. Virol J. 2012 Dec;9(1):78. doi: 10.1186/1743-422X-9-78 22490115 PMC3364910

[6] Khuri-Bulos N, Lawrence L, Piya B, Wang L, Fonnesbeck C, Faouri S, et al. Severe outcomes associated with respiratory viruses in newborns and infants: a prospective viral surveillance study in Jordan. BMJ Open. 2018 May;8(5):e021898. doi: 10.1136/bmjopen-2018-021898 29780032 PMC5961648

[7] Weber MW, Weber MW, Mulholland EK, Mulholland EK, Greenwood BM. Respiratory syncytial virus infection in tropical and developing countries. Trop Med Int Health. 1998 Apr;3(4):268–80. doi: 10.1046/j.1365-3156.1998.00213.x 9623927

[8] Picot VS, Bénet T, Messaoudi M, Telles JN, Chou M, Eap T, et al. Multicenter case–control study protocol of pneumonia etiology in children: Global Approach to Biological Research, Infectious diseases and Epidemics in Low-income countries (GABRIEL network). BMC Infect Dis. 2014 Dec;14(1):635. doi: 10.1186/s12879-014-0635-8 25927410 PMC4272811

[9] Emukule GO, Khagayi S, McMorrow ML, Ochola R, Otieno N, Widdowson MA, et al. The Burden of Influenza and RSV among Inpatients and Outpatients in Rural Western Kenya, 2009–2012. Aguilar PV, editor. PLoS ONE. 2014 Aug 18;9(8):e105543. doi: 10.1371/journal.pone.0105543 25133576 PMC4136876

[10] Talla Nzussouo N, Duque J, Adedeji AA, Coulibaly D, Sow S, Tarnagda Z, et al. Epidemiology of influenza in West Africa after the 2009 influenza A(H1N1) pandemic, 2010–2012. BMC Infect Dis. 2017 Dec;17(1):745. doi: 10.1186/s12879-017-2839-1 29202715 PMC5716025

[11] Movva N, Suh M, Reichert H, Hintze B, Sendak MP, Wolf Z, et al. Respiratory Syncytial Virus During the COVID-19 Pandemic Compared to Historic Levels: A Retrospective Cohort Study of a Health System. J Infect Dis. 2022 Aug 15;226(Supplement_2):S175–83. 35968868 10.1093/infdis/jiac220 PMC9377040

[12] Olsen SJ, Azziz-Baumgartner E, Budd AP, Brammer L, Sullivan S, Pineda RF, et al. Decreased influenza activity during the COVID-19 pandemic-United States, Australia, Chile, and South Africa, 2020. Am J Transplant Off J Am Soc Transplant Am Soc Transpl Surg. 2020 Dec;20(12):3681–5. doi: 10.1111/ajt.16381 33264506 PMC7753605

[13] Wan WY, Thoon KC, Loo LH, Chan KS, Oon LLE, Ramasamy A, et al. Trends in Respiratory Virus Infections During the COVID-19 Pandemic in Singapore, 2020. JAMA Netw Open. 2021 Jun 28;4(6):e2115973. doi: 10.1001/jamanetworkopen.2021.15973 34181015 PMC8239970

[14] Tang HJ, Lai CC, Chao CM. Changing Epidemiology of Respiratory Tract Infection during COVID-19 Pandemic. Antibiot Basel Switz. 2022 Feb 25;11(3):315. 10.3390/antibiotics11030315 PMC8944752 35326778

[15] Kim KR, Park H, Kim DR, Kim YJ. Changes in epidemiology of parainfluenza virus and respiratory syncytial virus infection during coronavirus disease 2019 pandemic in Korea. Clin Exp Pediatr. 2022 Jun 15;65(6):320–1. doi: 10.3345/cep.2021.01823 35287257 PMC9171459

[16] Agca H, Akalin H, Saglik I, Hacimustafaoglu M, Celebi S, Ener B. Changing epidemiology of influenza and other respiratory viruses in the first year of COVID-19 pandemic. J Infect Public Health. 2021 Sep;14(9):1186–90. doi: 10.1016/j.jiph.2021.08.004 34399190

[17] World Population Review—Sierra Leone. Available from: https://worldpopulationreview.com/countries/sierra-leone-population.

[18] United Nations Development Programme (UNDP). Human Development Index (HDI) 2022—Sierra Leone. Available from: https://hdr.undp.org/data-center/country-insights#/ranks.

[19] Samuels RJ, Moon TD, Starnes JR, Alhasan F, Gbakie M, Goba A, et al. Lassa Fever among Children in Eastern Province, Sierra Leone: A 7-year Retrospective Analysis (2012–2018). Am J Trop Med Hyg. 2021 Feb 3;104(2):585–92. 10.4269/ajtmh.20-0773 PMC7866338 33241780

[20] Shaffer JG, Schieffelin JS, Gbakie M, Alhasan F, Roberts NB, Goba A, et al. A medical records and data capture and management system for Lassa fever in Sierra Leone: Approach, implementation, and challenges. PloS One. 2019;14(3):e0214284. doi: 10.1371/journal.pone.0214284 30921383 PMC6438490

[21] World Health Organization. Global Influenza Programme. Influenza surveillance outputs. Available from: https://www.who.int/teams/global-influenza-programme/surveillance-and-monitoring/influenza-surveillance-outputs.

[22] Kebede S, Conteh IN, Steffen CA, Vandemaele K, Wurie I, Alemu W, et al. Establishing a national influenza sentinel surveillance system in a limited resource setting, experience of Sierra Leone. Health Res Policy Syst. 2013 Dec;11(1):22. 23800108 10.1186/1478-4505-11-22 PMC3694480

[23] Shaffer JG, Grant DS, Schieffelin JS, Boisen ML, Goba A, Hartnett JN, et al. Lassa Fever in Post-Conflict Sierra Leone. Bird B, editor. PLoS Negl Trop Dis. 2014 Mar 20;8(3):e2748. doi: 10.1371/journal.pntd.0002748 24651047 PMC3961205

[24] Shaffer JG, Schieffelin JS, Grant DS, Goba A, Momoh M, Kanneh L, et al. Data set on Lassa fever in post-conflict Sierra Leone. Data Brief. 2019 Apr;23:103673. doi: 10.1016/j.dib.2019.01.021 30788396 PMC6369334

[25] Hands C, Hands S, Verriotis M, Bunn J, Bailey E, Samuels RJ, et al. Emergency Triage Assessment and Treatment Plus (ETAT+): adapting training to strengthen quality improvement and task-sharing in emergency paediatric care in Sierra Leone. J Glob Health. 2021 Dec 18;11:04069. doi: 10.7189/jogh.11.04069 34956636 PMC8684797

[26] Tal A, Bavilski C, Yohai D, Bearman JE, Gorodischer R, Moses SW. Dexamethasone and salbutamol in the treatment of acute wheezing in infants. Pediatrics. 1983 Jan;71(1):13–8. 6129609

[27] Liu Q, Shepherd BE, Li C, Harrell FE. Modeling continuous response variables using ordinal regression: Modeling continuous response variables using ordinal regression. Stat Med. 2017 Nov 30;36(27):4316–35. 28872693 10.1002/sim.7433 PMC5675816

[28] Shepherd BE., Rebeiro PF., & the Caribbean, Central and South America network for HIV epidemiology. Brief Report: Assessing and Interpreting the Association Between Continuous Covariates and Outcomes in Observational Studies of HIV Using Splines. *JAIDS J*. *Acquir*. *Immune Defic*. *Syndr*. 2017 Mar 1;74(3):e60–e63. doi: 10.1097/QAI.0000000000001221 27798430 PMC5303133

[29] D’Agostino RB. Propensity score methods for bias reduction in the comparison of a treatment to a non-randomized control group. *Stat Med*. 1998 Oct 15;17(19):2265–81. doi: 10.1002/(sici)1097-0258(19981015)17:19&lt;2265::aid-sim918&gt;3.0.co;2-b 9802183

[30] Gupta S, Gupta T, Gupta N. Global respiratory virus surveillance: strengths, gaps, and way forward. Int J Infect Dis IJID Off Publ Int Soc Infect Dis. 2022 Aug;121:184–9. doi: 10.1016/j.ijid.2022.05.032 35584744 PMC9107382

[31] Neumann G, Kawaoka Y. Seasonality of influenza and other respiratory viruses. EMBO Mol Med. 2022;14(4). doi: 10.15252/emmm.202115352 35157360 PMC8988196

[32] Lopes GP, Amorim ÍPS, Melo B de O de, Maramaldo CEC, Bomfim MRQ, Lima Neto LG, et al. Identification and seasonality of rhinovirus and respiratory syncytial virus in asthmatic children in tropical climate. Biosci Rep. 2020 Sep 30;40(9):BSR20200634. doi: 10.1042/BSR20200634 32914848 PMC7517263

[33] Li Y, Reeves RM, Wang X, Bassat Q, Brooks WA, Cohen C, et al. Global patterns in monthly activity of influenza virus, respiratory syncytial virus, parainfluenza virus, and metapneumovirus: a systematic analysis. Lancet Glob Health. 2019 Aug;7(8):e1031–45. doi: 10.1016/S2214-109X(19)30264-5 31303294

[34] Lam TT, Tang JW, Lai FY, Zaraket H, Dbaibo G, Bialasiewicz S, et al. Comparative global epidemiology of influenza, respiratory syncytial and parainfluenza viruses, 2010–2015. J Infect. 2019 Oct;79(4):373–82. doi: 10.1016/j.jinf.2019.07.008 31323249 PMC7112594

[35] Paynter S. Humidity and respiratory virus transmission in tropical and temperate settings. Epidemiol Infect. 2015 Apr;143(6):1110–8. doi: 10.1017/S0950268814002702 25307020 PMC9507187

[36] Shi T, McAllister DA, O’Brien KL, Simoes EAF, Madhi SA, Gessner BD, et al. Global, regional, and national disease burden estimates of acute lower respiratory infections due to respiratory syncytial virus in young children in 2015: a systematic review and modelling study. The Lancet. 2017 Sep;390(10098):946–58. doi: 10.1016/S0140-6736(17)30938-8 28689664 PMC5592248

[37] Khuri-Bulos N, Williams JV, Shehabi AA, Faouri S, Jundi EA, Abushariah O, et al. Burden of respiratory syncytial virus in hospitalized infants and young children in Amman, Jordan. Scand J Infect Dis. 2010 Jan;42(5):368–74. doi: 10.3109/00365540903496544 20100116 PMC2854220

[38] Wu A, Mihaylova VT, Landry ML, Foxman EF. Interference between rhinovirus and influenza A virus: a clinical data analysis and experimental infection study. Lancet Microbe. 2020 Oct;1(6):e254–62. doi: 10.1016/s2666-5247(20)30114-2 33103132 PMC7580833

[39] Aung AH, Lye DC, Cui L, Ooi CK, Chow ALP. The “timeless” use of influenza-like illness criteria for influenza detection in the tropics. Int J Infect Dis. 2021 May;106:160–8. doi: 10.1016/j.ijid.2021.03.045 33741485

[40] Zheng X, Song Z, Li Y, Zhang J, Wang XL. Possible interference between seasonal epidemics of influenza and other respiratory viruses in Hong Kong, 2014–2017. BMC Infect Dis. 2017 Dec;17(1):772. 10.1186/s12879-017-2888-5 PMC5732536 29246199

[41] Bloom-Feshbach K, Alonso WJ, Charu V, Tamerius J, Simonsen L, Miller MA, et al. Latitudinal Variations in Seasonal Activity of Influenza and Respiratory Syncytial Virus (RSV): A Global Comparative Review. Cowling BJ, editor. PLoS ONE. 2013 Feb 14;8(2):e54445. doi: 10.1371/journal.pone.0054445 23457451 PMC3573019

[42] Azziz Baumgartner E, Dao CN, Nasreen S, Bhuiyan MU, Mah-E-Muneer S, Mamun AA, et al. Seasonality, Timing, and Climate Drivers of Influenza Activity Worldwide. J Infect Dis. 2012 Sep 15;206(6):838–46. doi: 10.1093/infdis/jis467 22829641

[43] Caini S, Andrade W, Badur S, Balmaseda A, Barakat A, Bella A, et al. Temporal Patterns of Influenza A and B in Tropical and Temperate Countries: What Are the Lessons for Influenza Vaccination? Schanzer DL, editor. PLOS ONE. 2016 Mar 31;11(3):e0152310. 10.1371/journal.pone.0152310 PMC4816507 27031105

[44] Stensballe LG, Devasundaram JK, Simoes EAF. Respiratory syncytial virus epidemics: the ups and downs of a seasonal virus: Pediatr Infect Dis J. 2003 Feb;22(Supplement):S21–32. 10.1097/01.inf.0000053882.70365.c9 12671449

[45] Tamerius JD, Shaman J, Alonso WJ, Bloom-Feshbach K, Uejio CK, Comrie A, et al. Environmental Predictors of Seasonal Influenza Epidemics across Temperate and Tropical Climates. Riley S, editor. PLoS Pathog. 2013 Mar 7;9(3):e1003194. doi: 10.1371/journal.ppat.1003194 23505366 PMC3591336

[46] Climate Change Adaptation Sierra Leone, United Nations Development Programme (UNDP). Available from: https://www.adaptation-UNDP.org/explore/western-africa/sierra-leone#.

[47] Groves HE, Piché-Renaud PP, Peci A, Farrar DS, Buckrell S, Bancej C, et al. The impact of the COVID-19 pandemic on influenza, respiratory syncytial virus, and other seasonal respiratory virus circulation in Canada: A population-based study. Lancet Reg Health—Am. 2021 Sep;1:100015. doi: 10.1016/j.lana.2021.100015 34386788 PMC8285668

[48] Garg I, Shekhar R, Sheikh AB, Pal S. Impact of COVID-19 on the Changing Patterns of Respiratory Syncytial Virus Infections. Infect Dis Rep. 2022 Jul 24;14(4):558–68. doi: 10.3390/idr14040059 35893478 PMC9394296

[49] Sullivan SG, Carlson S, Cheng AC, Chilver MB, Dwyer DE, Irwin M, et al. Where has all the influenza gone? The impact of COVID-19 on the circulation of influenza and other respiratory viruses, Australia, March to September 2020. Eurosurveillance [Internet]. 2020 Nov 26 [cited 2023 Feb 26];25(47). Available from: https://www.eurosurveillance.org/content/10.2807/1560-7917.ES.2020.25.47.2001847 10.2807/1560-7917.ES.2020.25.47.2001847 PMC7693168 33243355

[50] Halasa N, Williams J, Faouri S, Shehabi A, Vermund SH, Wang L, et al. Natural history and epidemiology of respiratory syncytial virus infection in the Middle East: Hospital surveillance for children under age two in Jordan. Vaccine. 2015 Nov;33(47):6479–87. doi: 10.1016/j.vaccine.2015.08.048 26314623 PMC7115487

[51] Probst V, Rankin DA, Haddadin Z, Hamdan L, Rahman HK, Yanis A, et al. Adenovirus Infection in Hospitalized Children with Acute Respiratory Infection in Jordan. Pediatr Infect Dis J. 2022 Apr;41(4):277–83. doi: 10.1097/INF.0000000000003423 35315822 PMC8943843

[52] Dagne H, Andualem Z, Dagnew B, Taddese AA. Acute respiratory infection and its associated factors among children under-five years attending pediatrics ward at University of Gondar Comprehensive Specialized Hospital, Northwest Ethiopia: institution-based cross-sectional study. BMC Pediatr. 2020 Dec;20(1):93. doi: 10.1186/s12887-020-1997-2 32111196 PMC7047350

[53] Sg Kumar, A Majumdar, Kumar V, Naik B, Selvaraj K, Balajee K. Prevalence of acute respiratory infection among under-five children in urban and rural areas of puducherry, India. J Nat Sci Biol Med. 2015;6(1):3. doi: 10.4103/0976-9668.149069 25810626 PMC4367064

[54] Breiman RF, Cosmas L, Njenga MK, Williamson J, Mott JA, Katz MA, et al. Severe acute respiratory infection in children in a densely populated urban slum in Kenya, 2007–2011. BMC Infect Dis. 2015 Dec;15(1):95. 25879805 10.1186/s12879-015-0827-x PMC4351931

[55] Famoroti T, Sibanda W, Ndung’u T. Prevalence and seasonality of common viral respiratory pathogens, including Cytomegalovirus in children, between 0–5 years of age in KwaZulu-Natal, an HIV endemic province in South Africa. BMC Pediatr. 2018 Dec;18(1):240. 30031377 10.1186/s12887-018-1222-8 PMC6054853

[56] Brini I, Guerrero A, Hannachi N, Bouguila J, Orth-Höller D, Bouhlel A, et al. Epidemiology and clinical profile of pathogens responsible for the hospitalization of children in Sousse area, Tunisia. Lin B, editor. PLOS ONE. 2017 Nov 17;12(11):e0188325. doi: 10.1371/journal.pone.0188325 29149199 PMC5693464

[57] Ruuskanen O, Lahti E, Jennings LC, Murdoch DR. Viral pneumonia. The Lancet. 2011 Apr;377(9773):1264–75. doi: 10.1016/S0140-6736(10)61459-6 21435708 PMC7138033

[58] Debiaggi M, Canducci F, Ceresola ER, Clementi M. The role of infections and coinfections with newly identified and emerging respiratory viruses in children. Virol J. 2012 Dec;9(1):247. doi: 10.1186/1743-422X-9-247 23102237 PMC3573994

[59] Tazinya AA, Halle-Ekane GE, Mbuagbaw LT, Abanda M, Atashili J, Obama MT. Risk factors for acute respiratory infections in children under five years attending the Bamenda Regional Hospital in Cameroon. BMC Pulm Med. 2018 Dec;18(1):7. 29338717 10.1186/s12890-018-0579-7 PMC5771025

[60] Nshimiyimana Y, Zhou Y. Analysis of risk factors associated with acute respiratory infections among under-five children in Uganda. BMC Public Health. 2022 Dec;22(1):1209. doi: 10.1186/s12889-022-13532-y 35715771 PMC9205046

[61] Ujunwa F, Ezeonu C. Risk factors for acute respiratory tract infections in under-five children in Enugu Southeast Nigeria. Ann Med Health Sci Res. 2014;4(1):95. doi: 10.4103/2141-9248.126610 24669339 PMC3952306

[62] Arifeen S, Black RE, Antelman G, Baqui A, Caulfield L, Becker S. Exclusive Breastfeeding Reduces Acute Respiratory Infection and Diarrhea Deaths Among Infants in Dhaka Slums. Pediatrics. 2001 Oct 1;108(4):e67–e67. doi: 10.1542/peds.108.4.e67 11581475

[63] World Vision. Breastmilk, the perfect first food for newborns for at least six months. Available from: https://www.wvi.org/stories/breastmilk-perfect-first-food-new-born-least-six-months

[64] Sierra Leone passes ground-breaking law protect breastfeeding. Available from: https://reliefweb.int/report/sierra-leone/sierra-leone-passes-ground-breaking-law-protect-breastfeeding#.

[65] Roth D. Acute lower respiratory infections in childhood: opportunities for reducing the global burden through nutritional interventions. Bull World Health Organ. 2008 May 1;86(5):356–64. doi: 10.2471/blt.07.049114 18545738 PMC2647440

[66] Pelletier DL, Frongillo EA, Habicht JP. Epidemiologic evidence for a potentiating effect of malnutrition on child mortality. Am J Public Health. 1993 Aug;83(8):1130–3. doi: 10.2105/ajph.83.8.1130 8342721 PMC1695164

[67] Ezzati M, World Health Organization, editors. Comparative quantification of health risks: global and regional burden of disease attributable to selected major risk factors. Geneva: World Health Organization; 2004. 1 p.

[68] Black RE, Allen LH, Bhutta ZA, Caulfield LE, de Onis M, Ezzati M, et al. Maternal and child undernutrition: global and regional exposures and health consequences. The Lancet. 2008 Jan;371(9608):243–60. doi: 10.1016/S0140-6736(07)61690-0 18207566

