# Supplementary material for: Respiratory virus surveillance in hospitalized children less than two-years of age in Kenema, Sierra Leone during the COVID-19 pandemic (October 2020- October 2021)
Source: PLoS One. 2023 Oct 10;18(10):e0292652. doi: 10.1371/journal.pone.0292652 (PMC10564235; doi:10.1371/journal.pone.0292652)
Supplement: Attachment — Submitted filename: Samuels_PlosOne_Response to editor_02.09.23 (1).docx [file pone.0292652.s002.docx]

September 2, 2023

PLoS One

Editorial Staff

Dear Editors,

We would like to thank you for your observation and useful suggestion.

In the submission portal, we have changed the data availability statement to “Yes, all data are fully available without restriction.” A deidentified data set has been uploaded to <https://osf.io/2peh6/files/osfstorage/6493215b67aff805b3ee0303>

Please do not hesitate to contact us if you have any further questions.

Sincerely,

Robert J. Samuels.

***# Comment***

 Thank you for providing the following data availability statement:

"No - some restrictions will apply;

The data are not publicly available due to privacy or ethical restrictions. De-identified individual data and statistical codes that supports the findings of this study are available from the corresponding author on reasonable request. The data requestor will need to sign a data access agreement."

We also note you have indicated the following:

"a deidentified data set has been uploaded to https://osf.io/2peh6/files/osfstorage/6493215b67aff805b3ee0303"

Before we proceed, please clarify if the minimal data set underlying your study is available in the OSF repository.

R: **This has been corrected in the submission portal**. **We will update your Data Availability statement on your behalf to reflect the information you provide.**

*R: a deidentified data set has been uploaded to* <https://osf.io/2peh6/files/osfstorage/6493215b67aff805b3ee0303>
